# Epstein-Barr Virus Induces Adhesion Receptor CD226 (DNAM-1) Expression during Primary B-Cell Transformation into Lymphoblastoid Cell Lines

**DOI:** 10.1128/mSphere.00305-17

**Published:** 2017-11-29

**Authors:** Lisa Grossman, Chris Chang, Joanne Dai, Pavel A. Nikitin, Dereje D. Jima, Sandeep S. Dave, Micah A. Luftig

**Affiliations:** a Department of Molecular Genetics and Microbiology, Center for Virology, Duke University Medical Center, Durham, North Carolina, USA; b Duke Cancer Institute and Department of Medicine, Center for Genomic and Computational Biology, Duke University Medical Center, Durham, North Carolina, USA; c Center of Human Health and The Environment and Bioinformatics Research Center, North Carolina State University, Raleigh, North Carolina, USA; UNC—Chapel Hill

**Keywords:** CD226, DNAM-1, Epstein-Barr virus, LMP1, NF-κB, adhesion molecules, lymphoma

## Abstract

Epstein-Barr virus (EBV), an oncogenic herpesvirus, infects and transforms primary B cells into immortal lymphoblastoid cell lines (LCLs), providing a model for EBV-mediated tumorigenesis. EBV transformation stimulates robust homotypic aggregation, indicating that EBV induces molecules that mediate cell-cell adhesion. We report that EBV potently induced expression of the adhesion molecule CD226, which is not normally expressed on B cells. We found that early after infection of primary B cells, EBV promoted an increase in CD226 mRNA and protein expression. CD226 levels increased further from early proliferating EBV-positive B cells to LCLs. We found that CD226 expression on B cells was independent of B-cell activation as CpG DNA failed to induce CD226 to the extent of EBV infection. CD226 expression was high in EBV-infected B cells expressing the latency III growth program, but low in EBV-negative and EBV latency I-infected B-lymphoma cell lines. We validated this correlation by demonstrating that the latency III characteristic EBV NF-κB activator, latent membrane protein 1 (LMP1), was sufficient for CD226 upregulation and that CD226 was more highly expressed in lymphomas with increased NF-κB activity. Finally, we found that CD226 was not important for LCL steady-state growth, survival in response to apoptotic stress, homotypic aggregation, or adhesion to activated endothelial cells. These findings collectively suggest that EBV induces expression of a cell adhesion molecule on primary B cells that may play a role in the tumor microenvironment of EBV-associated B-cell malignancies or facilitate adhesion in the establishment of latency *in vivo*.

**IMPORTANCE** Epstein-Barr virus (EBV) is a common human herpesvirus that establishes latency in B cells. While EBV infection is asymptomatic for most individuals, immune-suppressed individuals are at significantly higher risk of a form of EBV latent infection in which infected B cells are reactivated, grow unchecked, and generate lymphomas. This form of latency is modeled in the laboratory by infecting B cells from the blood of normal human donors *in vitro*. In this model, we identified a protein called CD226 that is induced by EBV but is not normally expressed on B cells. Rather, it is known to play a role in aggregation and survival signaling of non-B cells in the immune system. Cultures of EBV-infected cells adhere to one another in “clumps,” and while the proteins that are responsible for this cellular aggregation are not fully understood, we hypothesized that this form of cellular aggregation may provide a survival advantage. In this article, we characterize the mechanism by which EBV induces this protein and its expression on lymphoma tissue and cell lines and characterize EBV-infected cell lines in which CD226 has been knocked out.

## Abstract (precis)

Epstein-Barr virus (EBV) is a common human herpesvirus that establishes latency in B cells. While EBV infection is asymptomatic for most individuals, immune-suppressed individuals are at significantly higher risk of a form of EBV latent infection in which infected B cells are reactivated, grow unchecked, and generate lymphomas. This form of latency is modeled in the laboratory by infecting B cells from the blood of normal human donors *in vitro*. In this model, we identified a protein called CD226 that is induced by EBV but is not normally expressed on B cells. Rather, it is known to play a role in aggregation and survival signaling of non-B cells in the immune system. Cultures of EBV-infected cells adhere to one another in “clumps,” and while the proteins that are responsible for this cellular aggregation are not fully understood, we hypothesized that this form of cellular aggregation may provide a survival advantage. In this article, we characterize the mechanism by which EBV induces this protein and its expression on lymphoma tissue and cell lines and characterize EBV-infected cell lines in which CD226 has been knocked out.

## INTRODUCTION

The oncogenic human herpesvirus known as Epstein-Barr virus (EBV) infects over 90% of adults worldwide (1). EBV primarily infects B cells and epithelial cells and causes diseases ranging from infectious mononucleosis to malignancies, including endemic Burkitt’s lymphoma, Hodgkin’s lymphomas, and posttransplant lymphoproliferative disorders in B cells and nasopharyngeal carcinoma and gastric carcinoma in epithelial cells (2–4). As a model for lymphomagenesis, EBV is capable of transforming primary human B cells into lymphoblastoid cell lines (LCLs). LCLs follow a gene expression program known as “latency III,” which is characterized by the constitutive expression of six EBV nuclear antigens (EBNAs) and three latent membrane proteins, LMP1, -2A, and -2B, as well as over 40 viral small noncoding RNAs (5). Because the latency III program expressed by LCLs mirrors the gene expression of lymphomas in the immunosuppressed, LCLs provide a practical and accessible model for lymphomagenesis studies (5, 6).

During transformation, EBV induces broad changes in host gene expression driven by the expression of LMP1 roughly 2 to 3 weeks after infection (7). LMP1 mimics a constitutively activated CD40 receptor and provides signals from the cell membrane that activate the NF-κB pathway (8). In addition to altering cell growth and survival, expression of LMP1 and activation of the NF-κB pathway promote intercellular aggregation in LCLs, causing them to clump in culture (9). Interactions between LFA-1 (CD18/CD11a) and ICAM-1 (CD54), an NF-κB target, are critical to maintaining this constitutive aggregation (10, 11). Indeed, LCLs generated from patients with leukocyte adhesion deficiency (LAD), a rare immune disorder linked to defects in LFA-1, fail to aggregate in culture (12). Similarly, LCLs treated with simvastatin, which binds to and inhibits LFA-1, disaggregate, downregulate NF-κB activity, and eventually undergo apoptosis (13).

Interestingly, comparative microarray analysis of uninfected and EBV-transformed primary human B cells identified CD226, also known as DNAX accessory molecule-1 (DNAM-1), as another adhesion protein upregulated during transformation (7). CD226 is highly expressed in T lymphocytes and NK cells, where it associates with LFA-1 in the plasma membrane, stabilizing its interaction with ICAM-1 (14, 15). However, only 3% of B cells normally express CD226, making its presence in LCLs highly atypical (14). The present investigation characterizes the regulation of CD226 expression by EBV during primary B-cell infection and its role in cell growth and survival.

## RESULTS

### EBV induces CD226 expression during primary B-cell transformation into LCLs.

**FIG 1:**
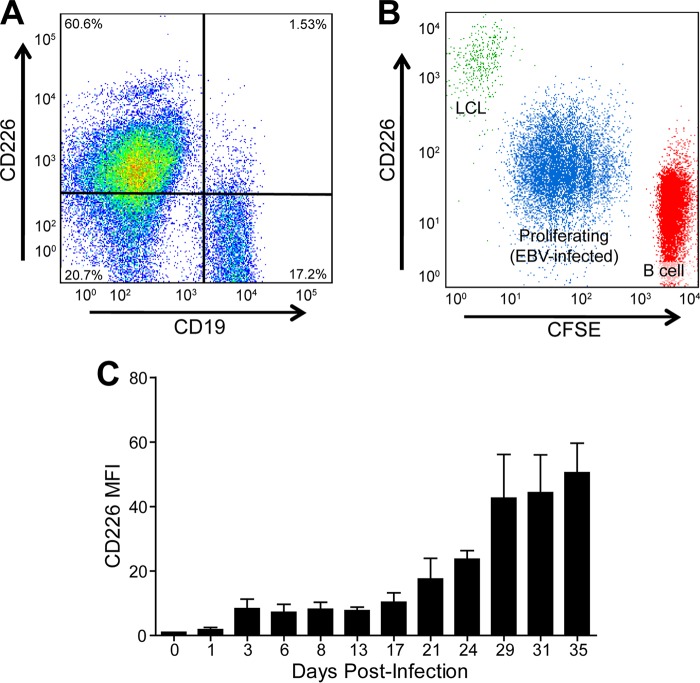
EBV upregulates CD226 during primary B-cell outgrowth into LCLs. (A) Representative flow cytometry plot of peripheral blood mononuclear cells (PBMCs). B cells are CD19^+^. (B) Overlaid CD226/CFSE flow cytometry plots of uninfected primary B cells (red), proliferating EBV-infected B cells (blue), and LCLs (green) from a matched biological donor. (C) CD226 flow cytometry of primary B cells at select days following EBV infection. The mean and standard deviation (SD) from two donors is shown.

We first sought to corroborate our previous finding that EBV induced CD226 expression upon primary B-cell infection (7). Peripheral blood mononuclear cells (PBMCs) were isolated, stained with anti-CD226 and anti-CD19, a B-cell marker, and analyzed by flow cytometry. CD19-negative cells (e.g., T and NK cells) exhibited high surface expression of CD226, while CD19-positive B cells exhibited low levels of CD226 expression (Fig. 1A). Next, donor-matched uninfected primary B cells, proliferating EBV-infected B cells, and LCLs were stained with anti-CD226 and carboxyfluorescein succinimidyl ester (CFSE), a dye that tracks cell proliferation. While less than 10% of uninfected primary B cells express CD226, CD226 expression increased significantly during proliferation and again during outgrowth into LCLs (Fig. 1B).

To more precisely define the time frame of CD226 expression during transformation, surface expression was tracked by flow cytometry across B cells from 2 donors following infection with EBV (Fig. 1C). CD226 levels increased roughly 5-fold from uninfected to day 3 (postinfection) and then remained stable through day 13. From day 13 to day 35, CD226 expression further increased, stabilizing at a 50-fold increase relative to uninfected B cells. The most dramatic increase in CD226 surface expression occurred approximately 3 weeks after infection, which is the same time frame that EBV induces NF-κB targets concomitant with LMP1 expression (7).

### CD226 induction is EBV specific and independent of B-cell activation.

**FIG 2:**
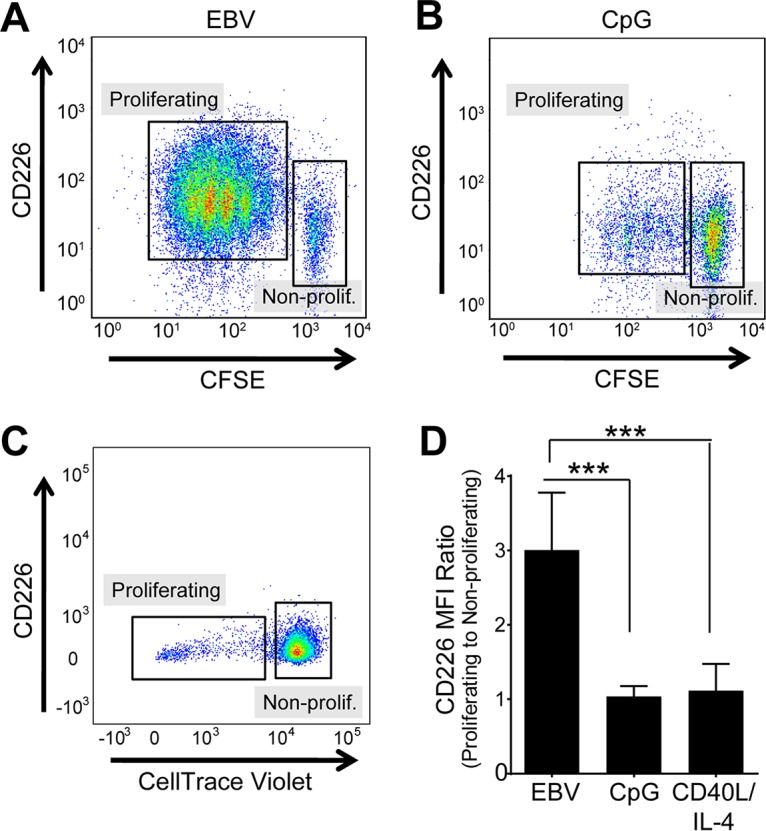
EBV, but not CpG DNA, upregulates CD226 upon cell proliferation. Representative flow cytometry plots of EBV-infected (A), CpG-activated (B), or CD40L/IL-4-activated B cells (C) at day 6 posttreatment/infection. Lower CFSE or CellTrace violet staining indicates proliferating cells. (D) CD226 mean fluorescence intensity (MFI) of proliferating population normalized to that of the nonproliferating population in EBV-infected (10 donors), CpG-activated (4 donors), and CD40L/IL-4-activated (4 donors) B cells. ***, *P* < 0.001 by one-way analysis of variance (ANOVA).

Because EBV mimics B-cell activation, we considered the possibility that B-cell activation induces CD226, and EBV does not specifically target CD226. To test this possibility, we activated B cells using CpG DNA, a pathogen-associated molecular pattern (PAMP) that stimulates the Toll-like receptor 9 (TLR9) pathway, and CD40L/interleukin-4 (IL-4), mitogens that activate B cells by mimicking T-cell-derived survival signals. CpG, CD40L/IL-4, and EBV infection induce proliferation in human B cells (Fig. 2A to C), but EBV-infected cells exhibited a roughly 3-fold increase in CD226 expression compared to mitogen-stimulated B cells, suggesting that CD226 upregulation is unique to EBV infection (Fig. 2D).

### EBV-positive, latency III lymphoblasts express higher CD226 levels than EBV-negative and EBV-positive latency I and Wp-restricted lymphoblasts.

**FIG 3:**
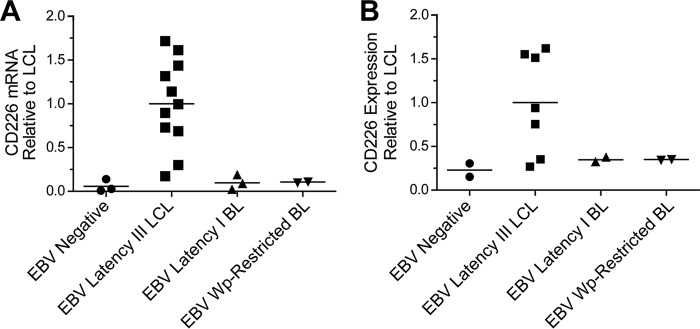
EBV-positive lymphoblasts express higher levels of CD226 than EBV-negative lymphoblasts. (A) qRT-PCR and (B) flow cytometry assessed CD226 mRNA and surface expression across a range of EBV-negative and EBV-positive (latency III, latency I, and Wp-restricted) B-lymphoblast (BL) cell lines.

Having verified that EBV specifically upregulates CD226 during transformation, we sought to determine whether CD226 is expressed across a broad range of EBV-positive B lymphoblasts. CD226 expression was measured at the mRNA level by quantitative reverse transcription-PCR (qRT-PCR) in EBV-negative B-cell lymphoma cell lines BJAB, BL41, and DG75, LCLs derived from 11 different donors, EBV-infected latency I Burkitt lymphoma cell lines Awia clone 9 and Rael, and Wp-restricted (EBNA2-deleted) Burkitt lymphoma cell lines Sal-BL and Oku-BL (16). CD226 surface expression was then analyzed by flow cytometry in BJAB, DG75, LCLs derived from 7 different donors, Awia clone 9, Rael, Sal-BL, and Oku-BL. EBV-negative lines, EBV latency I lines, and EBV Wp-restricted lines all expressed low levels of CD226 mRNA and surface protein (Fig. 3A and B). On average, EBV-negative lines expressed 17.2 times less CD226 mRNA than LCLs and 4.4 times less CD226 surface protein. Similarly, EBV latency I cell lines expressed 10.3 times less mRNA and 2.9 times less surface protein, while EBV Wp-restricted lines expressed 9.5 times less mRNA and 2.9 times less surface protein than LCLs. These data are consistent with a role for viral proteins unique to latency III in CD226 regulation.

### LMP1 and NF-κB activity are important for CD226 expression.

**FIG 4:**
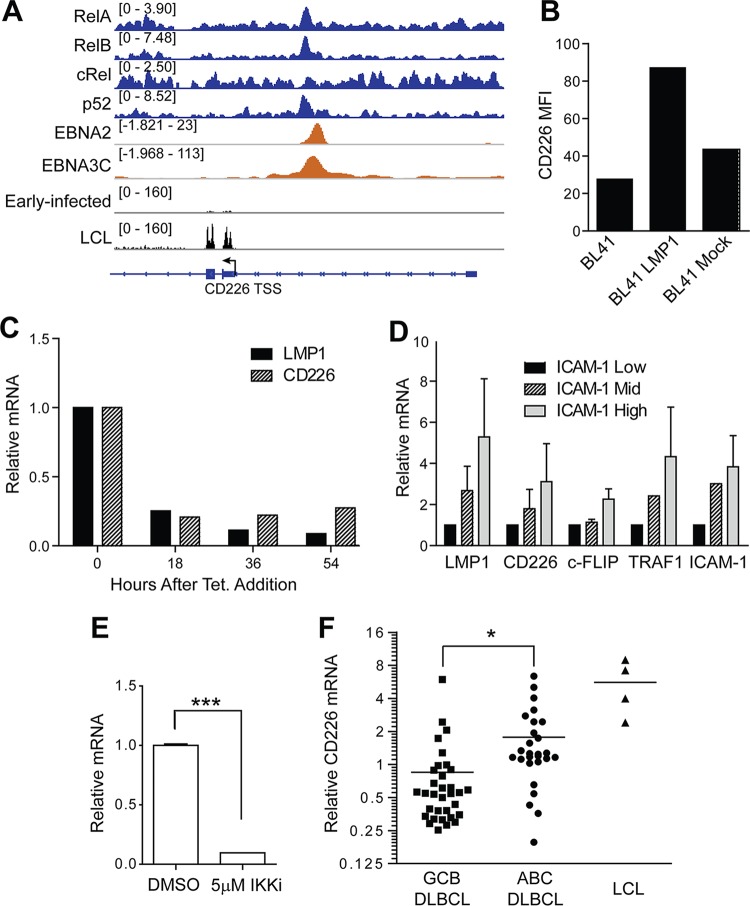
LMP1 and NF-κB regulate CD226 expression. (A) ChIP-seq of NF-κB transcription factors (RelA, RelB, cRel, and p52) and EBV nuclear antigens (EBNA2 and EBNA3C) at the CD226 locus. RNA-seq coverage at the CD226 locus is shown below from early-infected B cells and LCLs. (B) BL41 cells transduced with an LMP1-expressing retrovirus or mock transduced with an empty vector control and then assayed by flow cytometry for CD226 surface expression. (C) BL41 cells were transduced with tetracycline-responsive LMP1 and assayed for surface levels of CD226 and LMP1 expression by flow cytometry at 18-h intervals following treatment with tetracycline. (D) LCLs were sorted into low-, middle-, and high-ICAM-1-expressing populations using FACS. mRNA levels of CD226, LMP1, and NF-κB targets (TRAF1, ICAM-1, and c-FLIP) were determined by qRT-PCR from two independent experiments. Data are reported as means ± SD. (E) LCLs were treated with 5 μM IKKβ inhibitor IV and DMSO and then analyzed for CD226 RNA expression with qRT-PCR. Data are reported as means ± SD. (F) CD226 mRNA expression on GCB DLBCLs, ABC DLBCLs, and LCLs was determined using microarray analysis. *, *P* < 0.05; ***, *P* < 0.001.

The upregulation of CD226 mRNA and surface expression over the course of EBV-mediated outgrowth of *in vitro*-infected B cells corresponded strongly with the induction of the EBV nuclear antigens (EBNAs) and latent membrane protein 1 (LMP1), the viral oncoprotein that mimics a constitutively active CD40 to drive survival and proliferation through the NF-κB pathway (7). Chromatin immunoprecipitation sequencing (ChIP-seq) data from previously published work indicate that the NF-κB transcription factors (RelA, RelB, and p52) colocalize with EBNA2 and EBNA3C upstream of a putative alternative transcriptional start site (TSS) (17–20) (Fig. 4A). Furthermore, transcriptome sequencing (RNA-seq) analysis of early-infected B cells and LCLs confirms that the CD226 transcripts expressed in EBV-infected cells originate from the alternative TSS (Fig. 4A). These data suggest a landscape of regulation in which, early after infection, EBV transcriptional regulators (e.g., EBNA2 and EBNA3C) promote CD226 expression, which is then amplified in the LCL state in the presence of LMP1 signaling through NF-κB.

Because LMP1 is a major regulator of cell gene expression in latency III-expressing cells, we hypothesized that LMP1 activity was important for CD226 expression (10, 21). Therefore, we assessed the ability of LMP1 to induce CD226 using four distinct approaches. First, LMP1 was expressed in CD226-negative BL41 cells. We observed a 2-fold increase in CD226 surface expression following LMP1 transduction in BL41 cells relative to control transduced cells (Fig. 4B). Consistently, we found that CD226 mRNA levels were decreased following LMP1 depletion in BL41 cells stably expressing tetracycline-regulated LMP1 (Fig. 4C) (10). Finally, we assessed the levels of CD226 mRNA in LCLs sorted based on ICAM-1 expression. We have previously used ICAM-1 levels as a proxy of LMP1 and NF-κB activity within an LCL population (22). Here, we found that LMP1-low/ICAM-1-low LCLs displayed lower levels of CD226 mRNA than LMP1-high/ICAM-1-high LCLs (Fig. 4D). As controls, NF-κB targets TRAF1 and c-FLIP, as well as ICAM-1, were expressed at higher levels in ICAM-1-high-sorted cells than in ICAM-1-low-sorted cells (Fig. 4D).

In addition to our genetic approach to determine if LMP1 was required for CD226 expression, we treated LCLs with an IKKβ inhibitor. We observed a significant reduction in CD226 mRNA (Fig. 4E), indicating that NF-κB signaling downstream of LMP1 is important for CD226 expression at the transcriptional level. The linkage between CD226 expression and NF-κB in EBV-infected cells suggested that nonviral cancers with high NF-κB may also display elevated CD226 levels. To test this hypothesis, we evaluated CD226 mRNA expression in diffuse large B-cell lymphomas (DLBCLs) of the NF-κB-high, activated B-cell-like (ABC-DLBCL) and the NF-κB-low, germinal center B-cell-like (GCB-DLBCL) subsets. Consistent with our data in LCLs, we observed significantly higher CD226 mRNA levels in ABC-DLBCLs than GCB-DLBCLs (Fig. 4F) (23).

### Loss of CD226 does not impact LCL growth, susceptibility to apoptosis, homotypic aggregation, or binding to activated endothelial cells.

**FIG 5:**
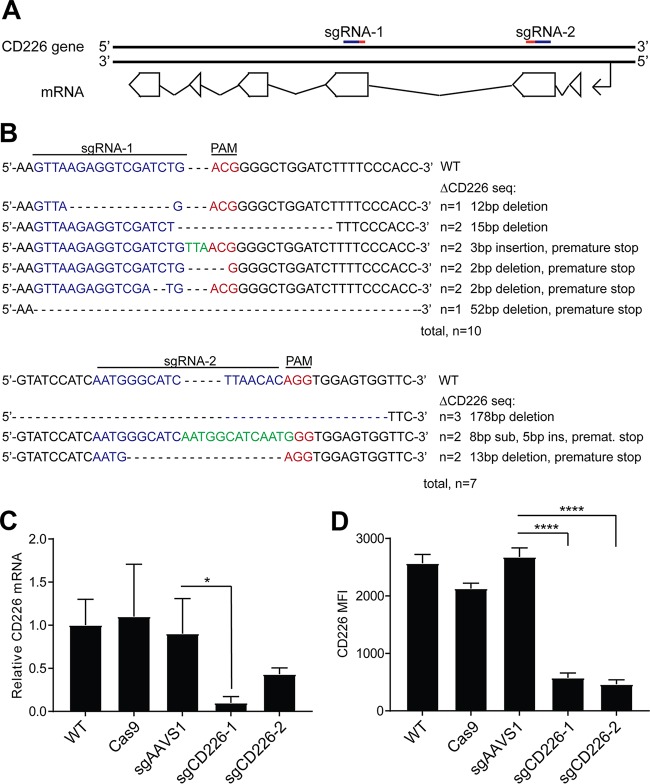
Generation of sgCD226 mutant LCLs using the CRISPR/Cas9 system. (A) Short guide RNAs (sgRNAs) were designed to target coding sequences of the CD226 gene. (B) Genetic lesions in the CD226 gene resulting from Cas9 cleavage and subsequent error-prone DNA repair were sequenced. The guide target is in blue, PAM is in red, and insertions are in green. (C) CD226 mRNA levels from the wild-type (WT) LCL, Cas9-only expressing LCL (Cas9), sgAAVS1-transduced Cas9-expressing LCL (sgAAVS1), and ΔCD226 LCLs (sgCD226-1 and sgCD226-2) were assayed by qRT-PCR from three independent experiments. Data are shown as means ± SD. *, *P* < 0.05 by Student’s *t* test. (D) The same cell lines in panel C were assayed for CD226 surface expression by flow cytometry in three independent experiments. Data are shown as means ± SD. ****, *P* < 0.0001 by Student’s *t* test.

To investigate the function of CD226 in LCLs, we utilized the clustered regularly interspaced short palindromic repeats system with Cas9 (CRISPR/Cas9) to impair its expression. We used two independently targeting synthetic short guide RNA (sgRNA) constructs to generate stable Cas9/sgRNA-expressing LCL lines, sgCD226-1 and -2 (Fig. 5A). We first confirmed the presence of lesions at the predicted target sites using Surveyor assays, and then we sequenced the genomic lesions generated by repair of the Cas9/sgRNA cleavage sites. The majority of clones sequenced had deletion and premature stop codons introduced by the repair process (Fig. 5B). Both CD226-knockout (sgCD226-1 and sgCD226-2) LCL lines displayed reduced mRNA, due to nonsense-mediated decay, and lower surface expression of CD226 relative to an LCL control expressing an sgRNA targeting the AAVS1 AAV integration site as well as a Cas9-only-expressing LCL and its wild-type (WT) LCL parent (Fig. 5C and D).

**FIG 6:**
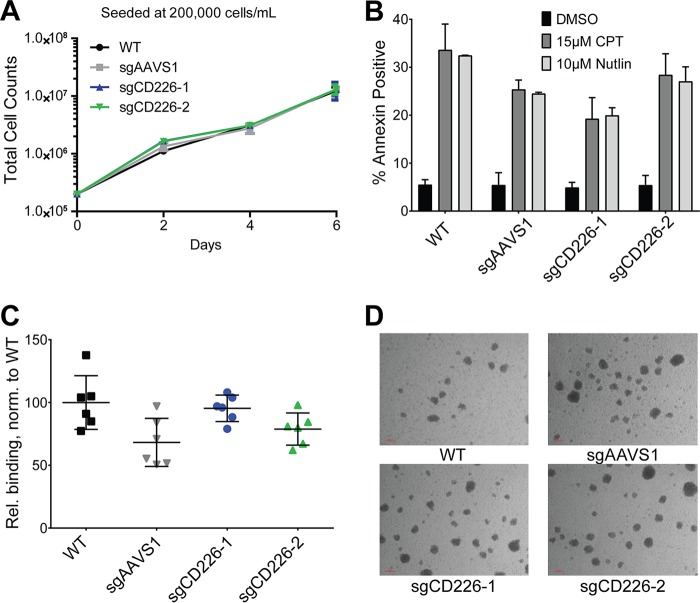
CD226 is not required for LCL proliferation or sensitivity to apoptosis relative to controls. (A) Growth of the WT LCL, sgAAVS1, and sgCD226-1 and -2 LCLs seeded at 200,000 cells/ml from three independent experiments. Data are shown as means ± SD. (B) The WT LCL, sgAAVS1, and sgCD226-1 and -2 LCLs were treated with Nutlin-3 (10 μM [two independent experiments]) or camptothecin (CPT; 15 μM [three independent experiments]) for 24 h. Apoptosis was quantified by flow cytometry for annexin V positivity. Data are reported as means ± SD. (C) Relative binding of WT, sgAAVS1, and sgCD226-1 and -2 LCLs to activated HUVECs from two independent experiments performed in triplicate. Data are reported as means ± SD. (D) Bright-field images of WT, sgAAVS1, and sgCD226-1 and -2 LCLs during culture (magnification, 40×).

We next assessed the growth and survival of LCLs lacking CD226. LCLs lacking CD226 seeded at a normal density of 200,000 cells/ml displayed equivalent growth rates over 6 days in culture to those LCLs expressing CD226 (Fig. 6A). To see if the absence of CD226 had an impact on cell survival, we treated control and sgCD226 LCLs with the topoisomerase inhibitor camptothecin (CPT) and the MDM2 antagonist Nutlin-3 to induce apoptosis. We observed robust annexin V positivity in all populations treated with both apoptotic inducers, indicating that CD226 was not critical for LCL survival (Fig. 6B). Finally, because previous work suggests a role for CD226 in adhesion, we measured the binding efficacy of wild-type and CD226 knockout LCLs to activated endothelial cells; however, in the absence of CD226, there was no effect of binding of LCLs to endothelial cells (Fig. 6C). Homotypic aggregation was also normal in the absence of CD226 as sgCD226 LCLs formed clumps of normal size during culture, as observed with a bright-field microscope (Fig. 6D).

## DISCUSSION

EBV-mediated transformation drives dramatic changes in gene expression by subverting the NF-κB signaling pathway through the activity of LMP1, which mimics a constitutively activated CD40 receptor (8). Increased NF-κB activity results in increased transcription and expression of adhesion proteins, such as LFA-1 and ICAM-1, which promote enhanced intercellular aggregation and survival (9, 24). In this report, we describe the EBV-induced expression of an additional adhesion protein, CD226, which has not been previously characterized in B cells or latent EBV infection.

Our data illustrate that CD226 follows an expression timeline and pattern similar to those of other adhesion proteins associated with EBV, such as ICAM-1 (7). Initially, B cells experience a minor boost in CD226 expression during EBV-induced proliferation. Then, roughly 3 weeks after infection, CD226 levels rapidly rise to the constitutively high levels of expression generally observed in LCLs. Additionally, like ICAM-1, CD226 is highly expressed in a majority of LCLs, which follow the latency III expression program, but is not expressed in lines following the less aggressive latency I program. Interestingly, CD40 ligation is insufficient in inducing CD226 expression in proliferating B cells, while the viral homologue of CD40, LMP1, sufficiently induces CD226 in the non-EBV Burkitt’s lymphoma line, BL41. This suggests a more complicated regulatory mechanism by which CD226 expression occurs in latency III LCLs, where viral transcriptional regulators cooperate with host transcriptional factors to coordinate targeted expression (25).

Although CD226 is minimally expressed and has no known function in B cells, similarities between adhesion in LCLs and NK and T cells suggest that CD226 may play an important role promoting intercellular interactions (14, 26, 27). Like LCLs, T lymphocytes and NK cells adhere to targets through interactions between LFA-1 and ICAM-1 (28). Cross-linking of LFA-1 then activates Fyn, a tyrosine kinase that phosphorylates CD226 and allows it to associate with LFA-1 in lipid rafts (29). Association with CD226 stabilizes the interaction between LFA-1 and ICAM-1 (24, 28). Indeed, endothelial ICAM-1 serves as a ligand for LFA-1 expressed on the surface of B cells, thereby facilitating endothelial cell-leukocyte interactions in lymphoid tissue (30). Finally, LFA-1 on the B cell and ICAM-1 on the epithelial cell are important for mediating EBV infection of epithelial cells; in fact, blocking LFA-1 or ICAM-1 with antibodies reduces the efficiency of infection (31). Alternatively, CD226 is not limited to its role in interactions between LFA-1 and ICAM-1. CD226 on the surface of NK and T cells can operate independently of LFA-1 through interactions with its ligands, CD155 and CD112, which influence cytotoxic T-cell and NK cell function and activation (32–34). In this way, CD226 expression in EBV-infected B cells may also play a novel role in B-cell activation.

Under many circumstances, expression of adhesion proteins is undesirable due to increased levels of immune detection. Both ICAM-1 and LFA-1 play critical roles in immune function and are downregulated in latency I lymphomas to assist immune evasion (35). However, in immunocompromised patients, such as those with posttransplant lymphoproliferative disease (PTLD) or AIDS-related malignancies, adhesion proteins are highly expressed (36). Interestingly, although enhanced aggregation and expression of adhesion proteins coincide with transformation, functional aggregation pathways are not directly required for transformation or growth in culture (12). By facilitating and stabilizing interactions between LFA-1 and ICAM-1, it may be that *in vivo*, heterotypic interactions with the surrounding stroma, mediated by CD226, may contribute to a survival and proliferative advantage for EBV-associated tumors.

## MATERIALS AND METHODS

### Cells and viruses.

Human peripheral blood mononuclear cells (PBMCs) were obtained using Ficoll purification (Sigma) from blood samples of EBV-seronegative anonymous donors from Carolina Red Cross (exempt from IRB review as nonhuman subject research). PBMCs were cultured in R15 complete medium (RPMI 1640 medium [Gibco] with 15% heat-inactivated fetal bovine serum [Gemini Bio], 0.5 µg/ml cyclosporine, 1 mM l-glutamine, 100 μg/ml penicillin-streptomycin) at 37°C and 5% CO_2_. B cells were isolated using BD IMag human B lymphocyte enrichment set (BD Biosciences) and cultured in R15 medium. The Awia clone 9, Rael, Sal-BL, and Oku-BL lines were cultured in R10 medium (RPMI 1640 medium [Gibco] with 10% heat-inactivated fetal bovine serum [Gemini Bio], 1 mM sodium pyruvate, 50 µM α-thioglycerol [Sigma], and 20 µM bathocuproine disulfonic acid [Sigma]). All other cell lines were cultured in R10 complete medium (RPMI 1640 medium [Gibco] with 10% heat-inactivated fetal bovine serum [Gemini Bio], 1 mM l-glutamine, 100 μg/ml penicillin-streptomycin) at 37°C and 5% CO_2_. Viable cell numbers were determined using a hemocytometer and trypan blue exclusion. B95-8 marmoset cells were cultured in R10 at 37°C and 5% CO_2_. Supernatants were filtered through a 0.45-μm-pore filter and used to infect PBMCs and B cells. The DG75, Awia clone 9, Rael, Sal-BL, and Oku-BL lines were received from the Andrew Bell Lab at University of Birmingham, United Kingdom. Tetracycline (Tet)-responsive BL41 was received from E. Cahir-McFarland and Elliott Kieff at Harvard Medical School (10).

### Generation of CRISPR lines.

An established LCL was transduced with the enhanced green fluorescent protein (EGFP) expression construct lentiCas9-EGFP (Addgene catalog no. 63592) and then subsequently transduced with one of three short guide RNAs (sgRNAs) expressed on the lentiguide-Puro vector (Addgene catalog no. 52963) to target the CD226 gene. Transduced cells were selected with puromycin and screened for efficiency of Cas9 cleavage by the Surveyor nuclease assay (37). Transduced cell lines were then assayed for CD226 mRNA and surface expression, of which two sgRNAs (sgCD226-1 and CD226-2) showed significantly reduced CD226 expression. Genetic lesions induced by Cas9 cleavage and error-prone DNA repair were sequenced by TOPO TA cloning (Invitrogen catalog no. 45003).

### Compounds.

All CpG experiments used CpG ODN 2006 ordered from Integrated DNA Technologies, Inc. (38). Human recombinant interleukin-4 (IL-4 [PeproTech catalog no. AF200-04]) was used at 20 ng/ml. Hemagglutinin (HA)-tagged CD40-ligand (R&D Systems catalog no. 6420-CL) was used at 5 ng/ml with an anti-HA cross-linking peptide (R&D Systems catalog no. MAB060) at 0.2 µg/µl. IKKβ inhibitor IV was used at 5 μM (EMD Millipore catalog no. 401481).

### EBV infection of cells.

Human PBMCs were infected as previously described (39). Briefly, 10^7^ PBMCs or 10^6^ B cells were infected with 500 μl filtered B95-8 Z-HT supernatant (multiplicity of infection [MOI] of 5) and incubated at 37°C for 1 h. Cells were subsequently resuspended in R15 medium at a concentration of 1 × 10^6^ cells per ml and used for further analysis.

### Flow cytometry.

Surface expression analysis of CD226 and CD19 was performed using the FACSCalibur (BD Biosciences) instrument. For labeling, cells were incubated with allophycocyanin (APC)-conjugated mouse anti-human CD226 antibody (Miltenyi Biotech, clone DX11), phycoerythrin (PE)-conjugated mouse anti-human CD226 antibody (BioLegend, clone 11A8), or PE-conjugated mouse anti-human CD19 antibody (BD Pharmingen) according to the manufacturer’s protocol. To observe proliferation, cells were stained with either 6-carboxyfluorescein succinimidyl ester (CFSE [Sigma; 21888]) or CellTrace Violet (CTV [Invitrogen; C34557]) according to the manufacturer’s protocol. Cell sorting was performed using the FACS-Diva (BD Biosciences) instrument.

### qRT-PCR.

To observe CD226 mRNA levels, total RNA was isolated from cells using the RNeasy minikit (Qiagen). Reverse transcription and cDNA amplification were done using the high-capacity cDNA reverse transcription kit (Applied Biosystems). RT-PCR was performed using the Quanta Perfecta SYBR green fast mix. Quantitative PCR (qPCR) results were normalized to SETDB-1. Primer sequences were as follows: CD226 forward, 5′-GGC AGA AGG TGA TAC AGG TG-3′; CD226 reverse, 5′-TCT TTT CCC ACC TCA CTG C-3′; ICAM-1 forward, 5′-ATG CCC AGA CAT CTG TGT CC-3′; ICAM-1 reverse, 5′-GGG GTC TCT ATG CCC AAC AA-3′; LMP1 forward, 5′-AAT TTG CAC GGA CAG GCA TT-3′; LMP1 reverse, 5′-AAG GCC AAA AGC TGC CAG AT-3′; SETDB-1 forward, 5′-TCC ATG GCA TGC TGG AGC GG-3′; SETDB-1 reverse, 5′-GAG AGG GTT CTT GCC CCG GT-3′; TRAF1 forward 5′-CCG GCC CCT GAT GAG AAT G-3′; TRAF1 reverse, 5′-TTC CTG GGC TTA TAG ACT GGA G-3′

### RNA-seq.

Four normal human donors were infected with EBV B95-8 (MOI of 5 as described above) and either sorted for CD19^+^/CTV^lo^ at day 7 postinfection or grown out into LCLs. RNA was extracted by Qiagen RNeasy, and libraries were made using the Kappa stranded mRNA-Seq kit (Kapa Biosystems catalog no. KR0960) with Illumina barcodes. Sequencing of these libraries was done at the Duke Genomics and Computational Biology Sequencing Core on the Illumina HiSeq4000. Data were analyzed using a standard pipeline as described in reference 40 aligning to the hg38 human genome. CD226 expression data were specifically extracted for one representative donor for early proliferating and LCL samples.

### Apoptosis experiments.

Cell lines were seeded at 500,000 cells/ml and treated with dimethyl sulfoxide (DMSO [untreated control, vehicle only]), 15 µM camptothecin (CPT), and 10 µM Nutlin-3 for 24 h. Samples were washed in 1× Annexin binding buffer and stained with APC-annexin V (BioLegend, catalog no. 640920) for 30 min to 1 h in the dark at 4°C and then washed in fluorescence-activated cell sorter (FACS) buffer (5% fetal bovine serum [FBS] in phosphate-buffered saline [PBS]) before analysis on a BD FACSCanto II.

### Endothelial cell binding assay.

Human umbilical vein endothelial cells (HUVECs) were a kind gift from Christopher Kontos at Duke University. HUVECs were seeded at 100,000 cells/well and activated with 100 ng/ml tumor necrosis factor alpha (TNF-α) (PeproTech catalog no. 300-01A) for 24 h prior to performing the assay. LCLs (WT, sgAAVS1, sgCD226-1, and sgCD226-2) were stained with calcein (Thermo, Fisher catalog no. C3100MP) and incubated with activated HUVECs at 37°C for 20 min. The plate was then imaged with the Cellomics ArrayScan VTI High Content (HC) cell-based imaging system at the Duke Functional Genomics Core and washed five times with PBS before imaging again. Relative binding was quantified by counting the number of calcein-positive events left remaining after the washes and by normalizing to that of WT LCLs.
